# Artificial intelligence for assessment of vascular involvement and tumor resectability on CT in patients with pancreatic cancer

**DOI:** 10.1186/s41747-023-00419-9

**Published:** 2024-02-12

**Authors:** Jacqueline I. Bereska, Boris V. Janssen, C. Yung Nio, Marnix P. M. Kop, Geert Kazemier, Olivier R. Busch, Femke Struik, Henk A. Marquering, Jaap Stoker, Marc G. Besselink, Inez M. Verpalen, for the Pancreatobiliary and Hepatic Artificial Intelligence Research (PHAIR) consortium

**Affiliations:** 1 Department of Radiology and Nuclear Medicine, Amsterdam UMC, Location University of Amsterdam, Amsterdam, The Netherlands; 2 Cancer Center Amsterdam, Amsterdam, The Netherlands; 3 Department of Biomedical Engineering and Physics, Amsterdam UMC, Location University of Amsterdam, Amsterdam, The Netherlands; 4 Department of Surgery, Amsterdam UMC, Location University of Amsterdam, Amsterdam, The Netherlands; 5 Department of Pathology, Amsterdam UMC, Location University of Amsterdam, Amsterdam, The Netherlands

**Keywords:** Artificial intelligence, Carcinoma (pancreatic ductal), Pancreatic neoplasms, Tomography (x-ray computed), Unsupervised machine learning

## Abstract

**Objective:**

This study aimed to develop and evaluate an automatic model using artificial intelligence (AI) for quantifying vascular involvement and classifying tumor resectability stage in patients with pancreatic ductal adenocarcinoma (PDAC), primarily to support radiologists in referral centers. Resectability of PDAC is determined by the degree of vascular involvement on computed tomography scans (CTs), which is associated with considerable inter-observer variability.

**Methods:**

We developed a semisupervised machine learning segmentation model to segment the PDAC and surrounding vasculature using 613 CTs of 467 patients with pancreatic tumors and 50 control patients. After segmenting the relevant structures, our model quantifies vascular involvement by measuring the degree of the vessel wall that is in contact with the tumor using AI-segmented CTs. Based on these measurements, the model classifies the resectability stage using the Dutch Pancreatic Cancer Group criteria as either resectable, borderline resectable, or locally advanced (LA).

**Results:**

We evaluated the performance of the model using a test set containing 60 CTs from 60 patients, consisting of 20 resectable, 20 borderline resectable, and 20 locally advanced cases, by comparing the automated analysis obtained from the model to expert visual vascular involvement assessments. The model concurred with the radiologists on 227/300 (76%) vessels for determining vascular involvement. The model’s resectability classification agreed with the radiologists on 17/20 (85%) resectable, 16/20 (80%) for borderline resectable, and 15/20 (75%) for locally advanced cases.

**Conclusions:**

This study demonstrates that an AI model may allow automatic quantification of vascular involvement and classification of resectability for PDAC.

**Relevance statement:**

This AI model enables automated vascular involvement quantification and resectability classification for pancreatic cancer, aiding radiologists in treatment decisions, and potentially improving patient outcomes.

**Key points:**

• High inter-observer variability exists in determining vascular involvement and resectability for PDAC.

• Artificial intelligence accurately quantifies vascular involvement and classifies resectability for PDAC.

• Artificial intelligence can aid radiologists by automating vascular involvement and resectability assessments.

**Graphical Abstract:**

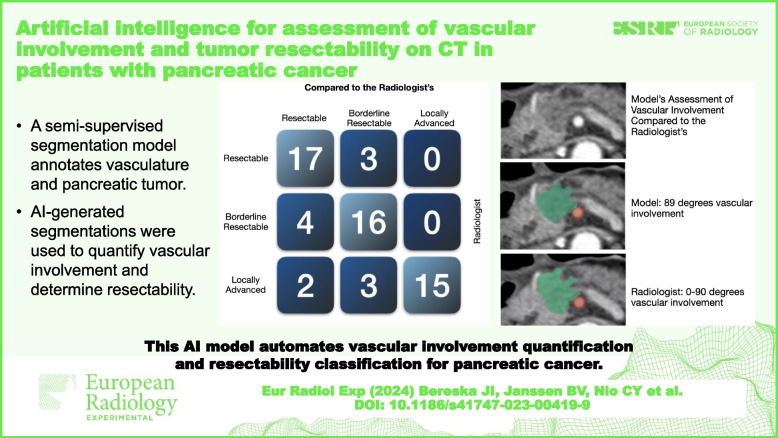

**Supplementary Information:**

The online version contains supplementary material available at 10.1186/s41747-023-00419-9.

## Background

Pancreatic ductal adenocarcinoma (PDAC) has a poor 5-year survival rate of less than 8% [1]. Surgical tumor removal combined with systemic therapy gives the best chances for long-term survival. The possibility of surgically removing a PDAC tumor depends on the extent of its involvement with several central blood vessels. Currently, radiologists use contrast-enhanced computed tomography scans (CTs) to determine the degree of vascular involvement. This assessment is subjective and prone to significant inter-observer variability, originating from difficulties in distinguishing PDAC from surrounding non-neoplastic tissue, including vessels and inflammatory infiltrates, especially for hypo-dense tumors [2, 3]. This variability can lead to suboptimal patient selection for surgery or studies and evaluation of outcomes, especially in referral centers with staff less experienced in PDAC assessment [2]. Artificial intelligence (AI) techniques may help address these issues and improve the variability of resectability assessments by aiding radiologists with an initial assessment.

Previous research on AI-based analysis of vascular involvement in PDAC has derived radiomic features to classify tumor resectability or evaluate contact between a particular vessel (such as the superior mesenteric artery [4], portal vein and superior mesenteric vein [5], superior mesenteric vein [6]) and the primary tumor [7]. However, these methods require manual segmentation of the tumor and surrounding vasculature, making them resource-intensive and impractical for clinical use. Additionally, since these models rely on hundreds of handcrafted radiomic features, the final decision of the model and its decision-making process is difficult to interpret, reproduce, and align with the current radiological workflow. Importantly, locally advanced PDAC (LAPC), *i.e*., PDAC with extensive vascular involvement deeming it unresectable, has not been included in previous computational approaches determining vascular involvement. Moreover, despite the increasing prevalence, no studies investigated patients treated with pre-operative systemic therapy. To address the limitations of these previous approaches, an automated and interpretable quantitative assessment of vascular involvement in patients with PDAC is needed. Deep learning techniques may meet this need.

In this study, we introduce an automatic UNet segmentation model for quantitatively assessing vascular involvement on CT. Our objectives are twofold: first to quantify vessel involvement in AI-generated segmentations of vasculature and tumor and second to leverage this quantification to determine PDAC resectability. Deep learning techniques are particularly suitable for this task due to their proficiency in handling complex patterns and large datasets, thereby potentially improving the reproducibility and efficiency of radiological assessments for vascular involvement and PDAC resectability.

## Methods

The Medical Ethics Review Committee of the Amsterdam UMC approved this study protocol and waived the need for informed consent. All patients were managed per institutional practices.

### Datasets

We used four datasets for this retrospective study containing 563 CTs of 467 patients with resectable, borderline resectable, and locally advanced PDAC, as well as CTs of control patients. The dataset characteristics are described in Table 1. The PREOPANC set is the Amsterdam UMC subset of the PREOPANC trials, randomized controlled trials performed by the Dutch Pancreatic Cancer Group (DPCG) [8, 9]. The LAPC set is a subset of patients from the DPCG LAPC registration [10]. The CONTROL set is a set of patients who received CTs prior to transcatheter aortic valve implantation and presented without pancreatic abnormalities. The primary purpose of the CONTROL set was to increase the volume of training data that depicts the entire pancreatic anatomy. By training the model on a more extensive and diverse set of pancreatic scans, we aimed to better capture the variabilities that naturally occur in the organ, thus enhancing the model’s accuracy and generalizability specifically for PDAC assessment. The MSKCC set is a publicly available dataset collected by the Memorial Sloan Kettering Cancer Center [11]. We selected late arterial phase CT scans (CT-LAPs) from patients in the PREOPANC, LAPC, and CONTROL datasets due to increased tumor visibility in this phase. Patients in these datasets received an intravenous injection of iodinated contrast agent (Xenetix®, 300 mg/mL, Guerbet, France, V08AB11). For patients in the MSKCC dataset*,* we used the portal-venous phase CT scans (CT-PVP), as this was the only available phase. The demographics and PDAC characteristics for the PREOPANC and LAPC datasets are presented in Table 2. General informed consent was obtained from all patients in the PREOPANC, LAPC, and CONTROL. Table 3 details the reconstruction and acquisition parameters used to obtain the CTs from all four datasets.

**Table 1:** Characteristics for all datasets

Characteristic	PREOPANC dataset	LAPC dataset	CONTROL dataset	MSKCC dataset
Number of scans	232	50	50	281
Number of patients	136	50	50	281
Timeframe	2013–2020	2019–2021	2013–2017	Not available
CT phase	Late arterial phase	Late arterial phase	Late arterial phase	Portal venous phase
Pancreatic abnormalities	Resectable and borderline resectable PDAC	Locally advanced PDAC	No pancreatic abnormalities	PDAC, intraductal mucinous neoplasms, pancreatic neuroendocrine tumors
Medical center	Amsterdam UMC	Amsterdam UMC	Amsterdam UMC	Memorial Sloan Kettering Cancer Center

**Table 2:** Patient demographics for the PREOPANC and LAPC datasets

Characteristic	PREOPANC dataset *N* = 232	LAPC dataset *N* = 50
Sex, *n* (%), female, male	57 (42), 79 (58)	76 (50.7), 74 (49.3)
Median age at diagnosis, years	66.1	64.4
Median tumor size, cm (dev)	3.02 (1.0)	4.3 (1.63)
Tumor location, *n* (%)		
Head	87 (68.5)	61 (48.8)
Uncinate	15 (11.8)	23 (18.4)
Body	11 (8.7)	29 (23.2)
Tail	14 (11)	12 (9.6)
Resection margin, *n* (%), R0, R1	65 (47.8), 33 (24.3)	13 (8.6), 7 (4.6)
Neoadjuvant therapy, *n* (%)		
None	35 (26.7)	125 (83.7)
FOLFIRINOX	32 (24.4)	25 (16.3)
Gemcitabine + radiation	64 (48.9)	0 (0)

*dev*, deviation; *R0*, radical resection; *R1*, non-radical resection; *FOLFIRINOX*, bolus and infusional 5-fluorouracil, leucovorin, irinotecan, and oxaliplatin; *Gemcitabine*, 2′-deoxy-2′,2′-difluorocytidine monohydrochloride (β isomer)

**Table 3:** Reconstruction and acquisition parameters used to obtain the CTs

Parameter	PREOPANC dataset *N* = 232	LAPC dataset *N* = 50	CONTROL dataset *N* = 50	MSKCC dataset *N* = 281
Pitch/table speed	0.6–0.938/15.64–61.4 mm	0.5–1.356/19.2–185 mm	0.914–1.9/134.3–437.6 mm	0.98–1.375/39.37–27.5 mm
x-ray tube current	50–484 mA	50–479 mA	77–2,551 mA	220–380 mA
kVp	100–120 kVp	80–120 kVp	70–120 kVp	120 kVp
Tube rotation speed	0.5–0.6 ms	0.4–0.5 ms	0.4 ms	0.7–0.8 ms
Slice thickness	1.0–5.0 mm	0.625–5.0 mm	1.0–3.0 mm	2.5 mm
Width × height	512–1,189 × 512–1,228	512–1,043 × 512–1,067	512 × 512	512 × 512
Machine manufacturer and type	SIEMENS: SOMATOM Force/Definition AS + /Edge/Flash, Aquilion ONE, iCT 256, Sensation64, Brilliance64 GE Healthcare: LightSpeed16, BrightSpeed Philips: Optima CT660	SIEMENS: SOMATOM Force/Definition AS + /Definition Edge, Aquilion ONE, iCT 256, Biograph64, Brilliance40/64, Philips: Ingenuity Core, Optima CT660	SIEMENS: SOMATOM Force/Definition AS + , iCT 256, Brilliance64	GE Healthcare

### Data preparation

Seven trained observers independently manually segmented anatomical structures in 105 CT-LAPs of 50 patients with (borderline) resectable PDAC (PREOPANC dataset), five patients with LAPC (LAPC dataset), and 50 control patients (CONTROL dataset) using 3D slicer version 4.11.20210226 [12]. The observers segmented the primary PDAC tumors and all five relevant vessels for determining PDAC resectability: celiac trunk (CeTr), hepatic artery (HA), portal vein (PV), superior mesenteric artery (SMA), and superior mesenteric vein (SMV) [12]. Including surrounding abdominal structures has been shown to increase model performance; therefore, they also segmented the pancreas, duodenum, liver, gallbladder, bile duct (and, if present, bile duct stents), aorta, splenic artery, splenic vein, and inferior vena cava [13]. The PDAC tumors were segmented by one of a team of three abdominal radiologists (C.Y.N., 27 years’ experience; F.S., 3 years’ experience; and M.P.M.K., 6 years’ experience) subspecialized in PDAC at the Amsterdam UMC. Subsequently, one of the three abdominal radiologists independently assessed the degree of vascular involvement by the tumor for the five relevant vessels. After the PDAC tumor was segmented, the remaining structures were segmented by one Ph.D. researcher (J.B.) and three Master researchers under the supervision of a radiology resident (I.V.) who also checked and, if needed, corrected the segmentations. Detailed information on the segmentation techniques used in 3D Slicer is provided in the Supplemental material.

### Model implementation

The overall workflow of our model consists of three automated steps: (1) segmenting the PDAC and vessels; (2) quantifying vascular involvement for CeTr, HA, SMA, SMV, and PV; and (3) classifying resectability.

#### Segmenting PDAC and vessels

We used a self-learning-based segmentation model for the PDAC and vessel segmentation; see Fig. 2 for a schematic representation. *Self-learning* is a two-stage learning paradigm in which a so-called *teacher segmentation model* is first trained on a small number of manually segmented training data. Subsequently, this teacher segmentation model is used to generate segmentations of the PDAC and vessels for the remaining training data. These segmentations are then used to train the *student segmentation model*. This student segmentation model constitutes the final segmentation model. This setup assumes that a student segmentation model trained on weak and noisy segmentations created by the teacher segmentation model can eventually surpass the teacher segmentation model’s performance by leveraging the additional data available for training [14]. In this manner, training with a self-learning paradigm can reduce the required manual segmentations and increase the segmentation model’s robustness and generalizability.

We trained the *teacher segmentation model* using 105 CT-LAPs of 50 patients with (borderline) resectable PDAC (PREOPANC dataset), five CT-LAPs of patients with LAPC (LAPC dataset), and 50 CT-LAPs of control patients (CONTROL dataset) with manual segmentations of the PDAC tumor, CeTr, HA, SMA, PV, SMV, pancreas, duodenum, liver, gallbladder, bile duct (and, if present, bile duct stents), aorta, splenic artery, splenic vein, and inferior vena cava. Subsequently, we used the teacher segmentation model to segment the remaining dataset of 508 CTs of controls and patients with PDAC. We then trained the *student segmentation model* using the resulting 508 AI-segmented scans generated by the teacher segmentation model and the 105 manually segmented scans. Lastly, we used the student segmentation model to segment the structures in the test dataset containing 60 randomly selected CT-LAPs from 60 patients from the PREOPANC and LAPC datasets that were not used to train the teacher segmentation model.

**Fig. 1:**
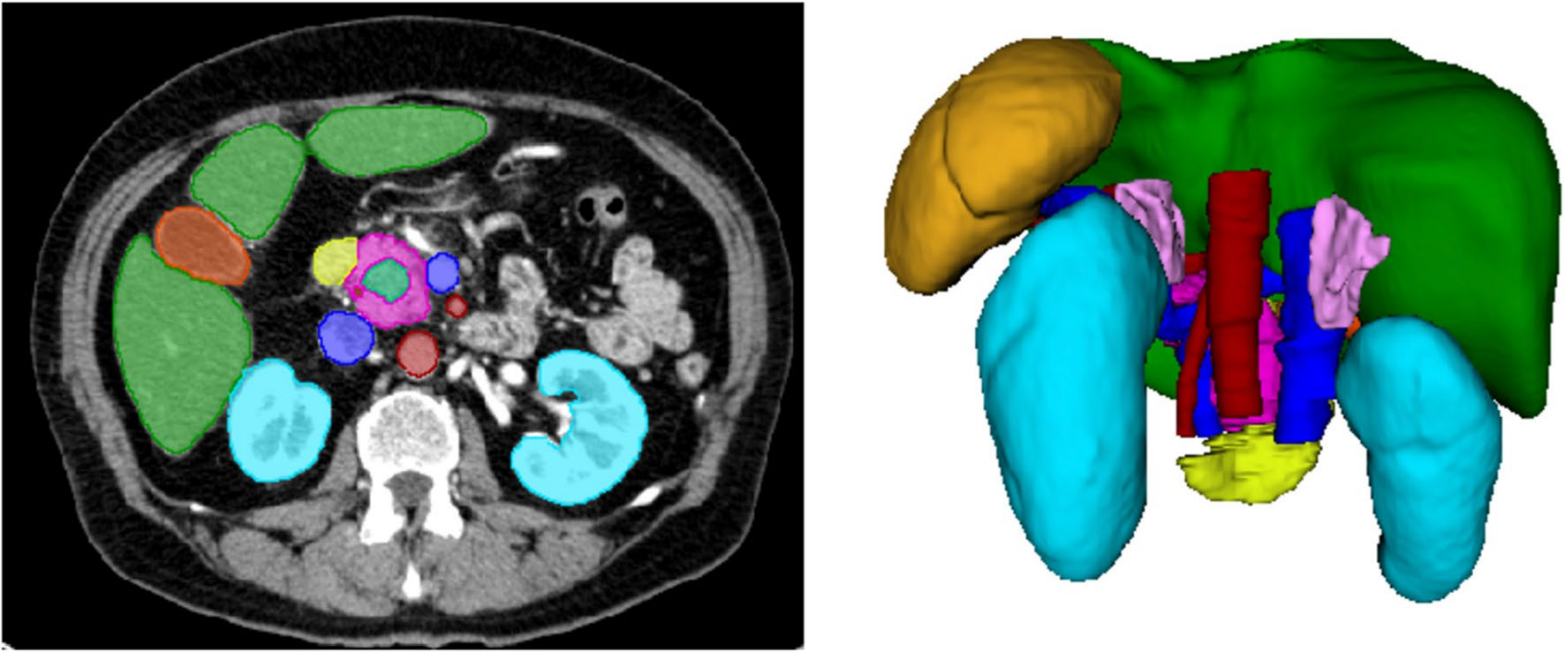
Example of a manual segmentation of anatomical structures within a late-arterial phase computed tomography scan. Turquoise = PDAC tumor; red = arteries (aorta, celiac trunk, hepatic artery, splenic artery, and superior mesenteric artery); dark blue = veins (inferior vena cava, portal vein, splenic vein, and superior mesenteric vein); pink = pancreas; yellow = duodenum; green = liver; orange = gallbladder. *PDAC*, Pancreatic ductal adenocarcinoma

**Fig. 2:**
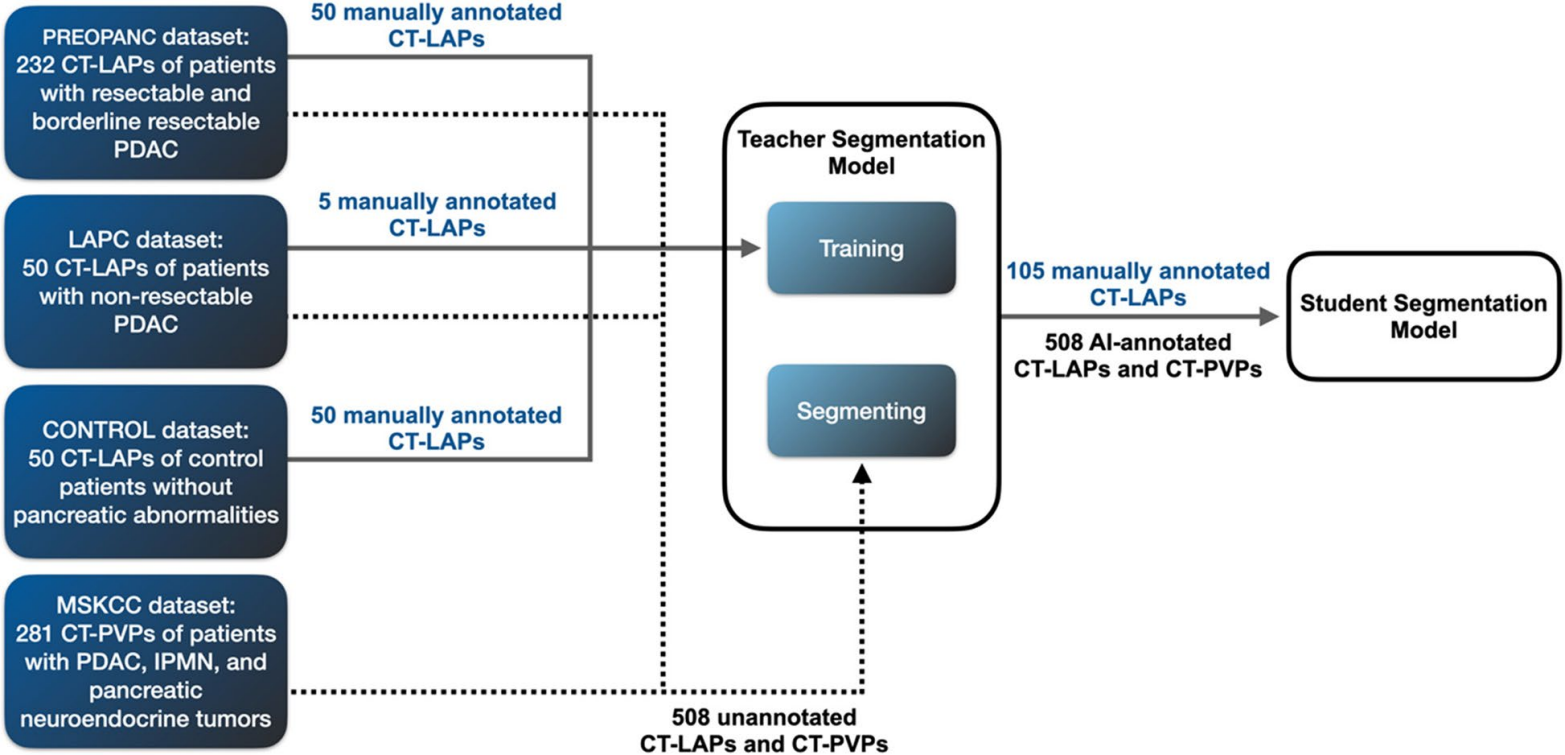
The proposed learning framework for PDAC, abdominal organ, and vasculature segmentation on contrast-enhanced CT scans. The teacher segmentation model was trained with 55 manually segmented CT-LAPs of patients with (borderline) resectable and locally advanced PDAC and 50 manually segmented CT-LAPs of control patients. The teacher segmentation model segments the remaining 458 CT-LAPs and CT-PVPs, which together with the 105 manually segmented CT-LAPs, were used to train the student segmentation model. The student segmentation model produced the final segmentations needed to determine vascular involvement and local tumor resectability. *CT*, Computed tomography; *CT-LAP*, Late arterial phase CT scan; *CT-PVP*, Portal venous phase CT scan; *PDAC*, Pancreatic ductal adenocarcinoma

We selected a nnUNet network setup consisting of a two-stage 3D U-Net cascade for both the student and the teacher segmentation model [15, 16]. The first U-Net was trained on down-sampled images and generated low-resolution segmentations, which serve as an auxiliary input for training the full-resolution U-Net. We used fivefold cross-validation with 1,000 steps per fold to train both the low-resolution and full-resolution U-Net. We trained all models on an NVIDIA A100 GPU, requiring approximately one day per fold. Figure 1 depicts an example of segmented CT-LAP from the training data. Figure 2 illustrates the training sets used for the teacher and student segmentation model.

#### Quantifying vascular involvement

To quantify the degree of involvement of a specific vessel by the PDAC tumor using the AI-generated 3D segmentations, we evaluated all x, y, and z planes containing both the vessel and PDAC. For each vessel, we determined the circumference of the vessel segment as well as the length of its connection with the PDAC tumor on every plane. The degree of involvement for each plane is defined as the following:

$$TV/\left(TV+ \left(TV-V\right)\right)\times 360$$

where *V* is the circumference of the vessel segment and *TV* is the length of its connection with the PDAC tumor. We finally selected the maximum degree of involvement over all planes for each vessel. The model quantified the degree of vascular involvement as a continuous variable with values ranging from 0 to 360.

#### Determining resectability stage

We followed the national guidelines provided by the Dutch Pancreatic Cancer Group (DPCG) in the definition of PDAC resectability [17]. The DPCG proposes four categories: resectable, borderline resectable, locally advanced, and metastasized PDAC. Metastasized PDAC is considered locally advanced regardless of vascular involvement. Patients with metastasized PDAC were excluded from this study as, in these cases, vascular involvement is not relevant to determine resectability. Additional file 1: Table S1 outlines the DPCG definitions for the remaining three categories. With these guidelines and the degrees of involvement obtained by the vessel involvement quantification measure, our model classifies tumors as resectable, borderline resectable, or locally advanced PDAC based on the measured degree of involvement.

### Performance assessment and statistical analysis

We compared the model’s assessment of vascular involvement with the assessment provided by one of three radiologists (C.Y.N., F.S., and M.P.M.K.). The radiologists’ categorized the degree of vascular involvement using five groups: 0, 0–90, 90–180, 180–270, and 270–360°. To align our model’s continuous output (ranging from 0 to 360°) with these categories, we transformed its assessments into the same categorical format. We then employed “agreement” as the primary metric for evaluating model performance, which signifies the number of vessels where the model’s categorization concurred with the radiologists.

For a more comprehensive understanding, we provided a visual representation of the distribution of vascular involvement assessed by the model per group. We further conducted a one-way analysis of variance (ANOVA) test to determine if there is a statistically significant difference between two or more of the groups with regard to the quantification provided by the model. A *p*-value of less than 0.05 was considered statistically significant.

We used agreement as a metric to evaluate the model’s performance for classifying resectability. Agreement measures the number of cases in which the model and the radiologist reached the same conclusion.

## Results

### Patient characteristics

The test set contained 60 CT-LAPs from 60 patients with resectable (20 CT-LAPs), borderline resectable (20 CT-LAPs), and locally advanced PDAC (20 CT-LAPs) of the PREOPANC and LAPC datasets. The test set comprised 28 females (47%) and 32 males (53%) with a median tumor diameter of 3.0 cm (standard deviation of 1.7 cm). Additional file 1: Table S2 details the distribution of vascular involvement in the test set for each of the five vessels considered.

### Quantifying vascular involvement

**Fig. 3:**
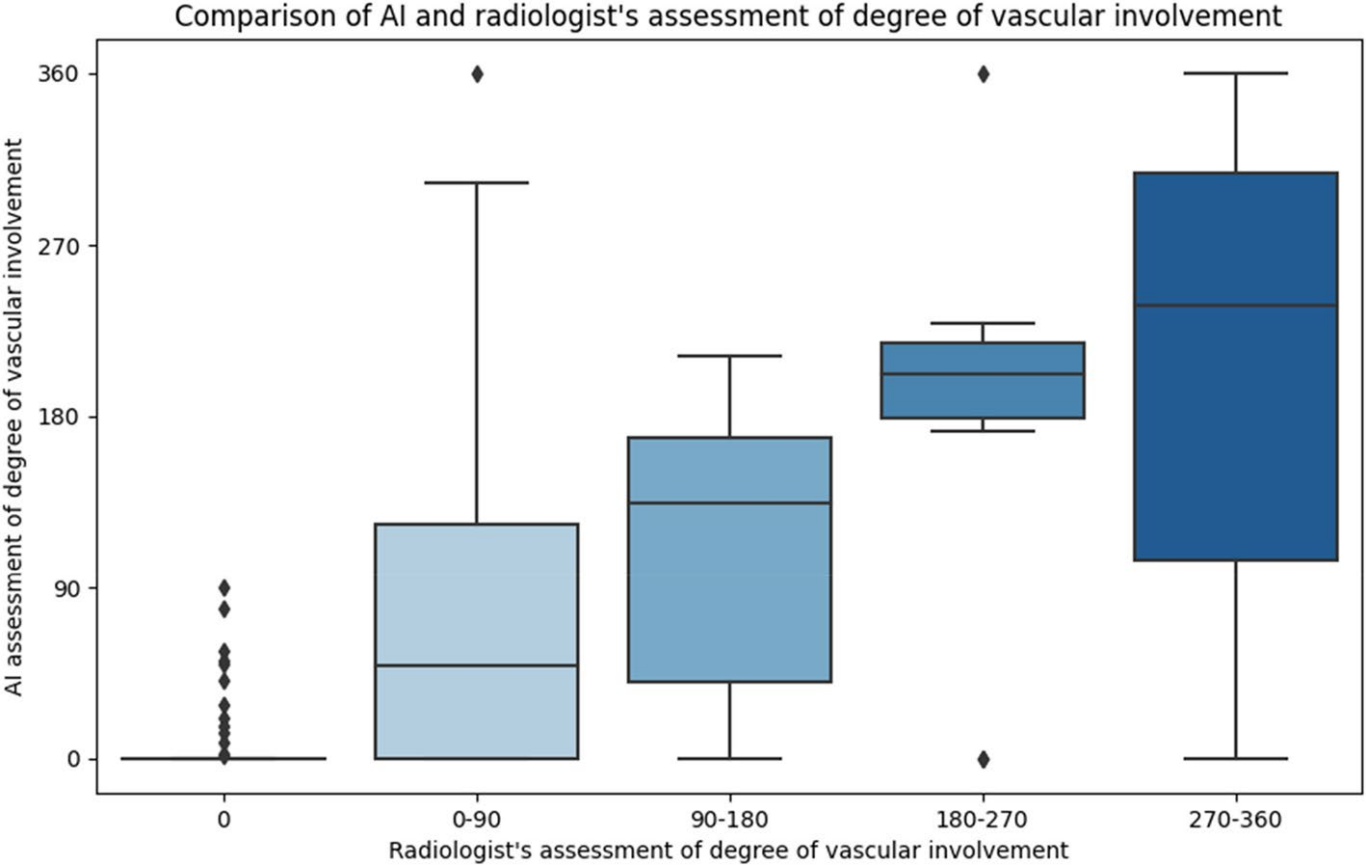
AI assessment of vascular involvement compared to radiologist’s assessment. The median is shown by the line that divides the box into two parts. The box represents the middle 50% of scores for that group. The upper and lower whiskers represent scores outside the middle 50%. Outliers with a score below or above 1.5 times the upper quartile are represented by the diamond mark

Our model demonstrated an overall agreement of 227/300 (76%) with the radiologist in quantifying vascular involvement from AI-generated CT segmentations in the 300 vessels of the 60 patients of the test set. When examining specific vessel types, the model achieved an agreement of 156/180 (87%) for arteries and 71/120 (59%) for veins. The vessel involvement quantified by the model increased with increasing involvement assessment by the radiologist. Specifically, the model’s median assessments for each group closely matched the expected values given the radiologists’ assessments. This relationship between the model’s assessment and the radiologist’s assessment of vascular involvement is described in Table 4. These results are further illustrated in Fig. 3. The one-way ANOVA revealed that there was a statistically significant difference in the degree of involvement between at least two groups (*p* = 10^−35^ <  < 0.05).

**Table 4:** Median assessment of vascular involvement by the model for each group assessed by the radiologist

Radiologist’s assessment of vascular involvement	Model’s median assessment of vascular involvement	IQR (Q1–Q3)
0	0	0 (0–0)
0–90	49	123 (0–43)
90–180	134	128 (40–167)
180–270	202	39 (179–218)
270–360	239	202 (104–307)

*IQR*, Interquartile range; *Q1*, Median of the lower half of the data; *Q3*, Median of the upper half of the data

**Fig. 4:**
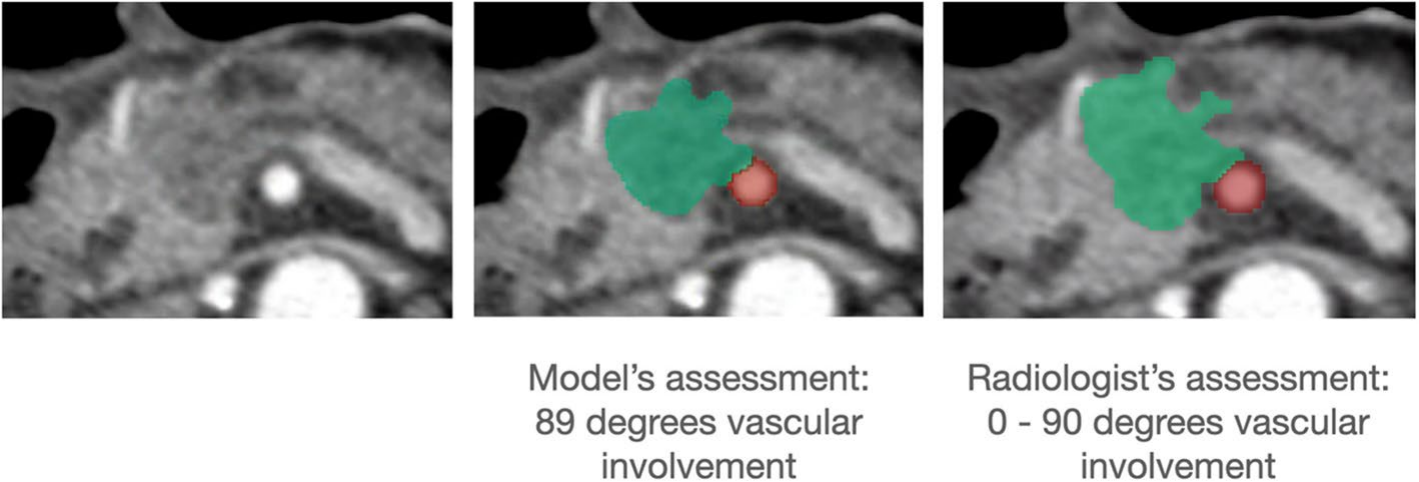
Example of a manually and an artificial intelligence (AI)-segmented computed tomography late arterial phase scan with the PDAC and superior mesenteric artery. Green = PDAC tumor; red = superior mesenteric artery. Using the AI-generated segmentation, our model quantifies the vascular involvement of the superior mesenteric artery by the PDAC tumor at 89°, which is in line with the radiologist’s assessment (0–90°). *PDAC*, Pancreatic ductal adenocarcinoma

Figure 4 gives an example of an AI-segmented scan with the PDAC tumor and an SMA and the model’s vascular involvement assessment, along with the manually segmented scan and vascular involvement assessment provided by the radiologist.

### Classifying resectability

**Fig. 5:**
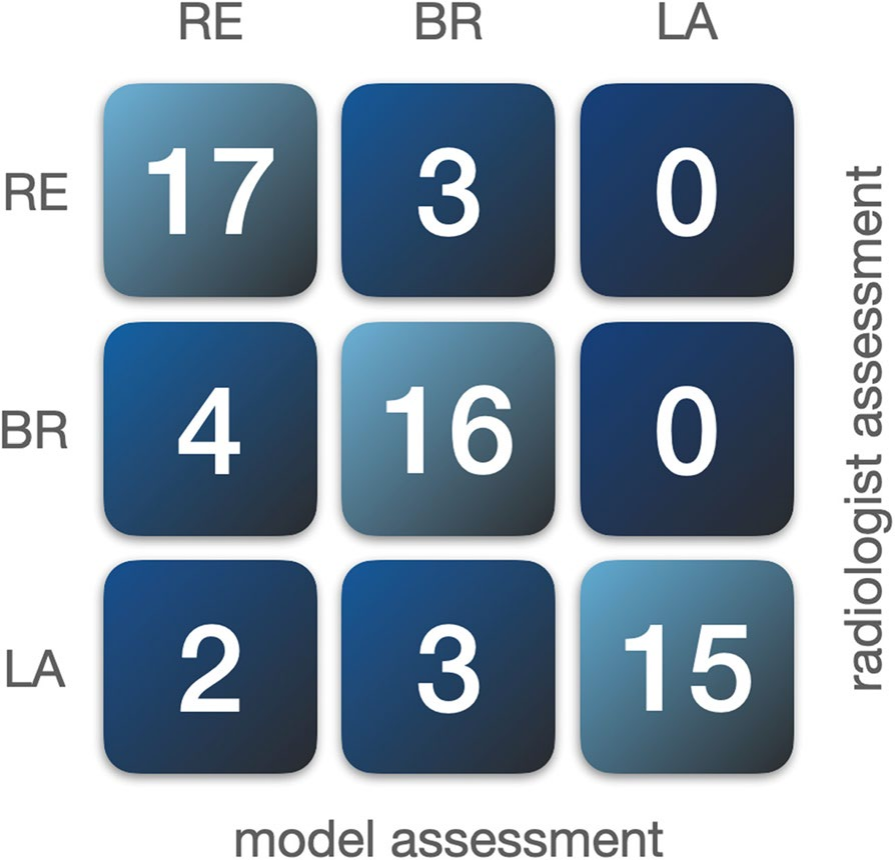
Agreement of our model with radiologist’s judgment for classifying tumor resectability. *RE*, Resectable pancreatic ductal adenocarcinoma (PDAC); *BR*, Borderline resectable PDAC; *LA*, Locally advanced PDAC

Our model obtained an overall agreement of 48/60 (80%) with the radiologist in classifying resectability. Our model classified resectable PDAC with an agreement of 17/20 (85%). For borderline resectable and locally advanced PDAC, similar scores of 16/20 (80%) and 15/20 (75%), respectively, were achieved. Figure 5 illustrates model performance by showing how often the model correctly classified resectability compared to the radiologist.

## Discussion

This is the first study to present a fully automated AI-based model for segmenting PDAC, quantifying vascular involvement, and determining resectability. The model achieved good agreement with the radiologists for assessing vascular involvement. Specifically, the mean value of vascular involvement, as measured by the model, demonstrated a strong relationship with the assessment provided by the radiologist. Additionally, the model classified tumor resectability with a strong agreement with the radiologist. These findings suggest that the proposed model has the potential to provide reliable and consistent measurements of vascular involvement and tumor resectability in PDAC patients.

Three previous studies have described models for classifying vascular involvement in patients with PDAC. While our model is capable of assessing vascular involvement for all five relevant vessels, previous studies have only examined single vessel segments (*e.g.*, SMA [4], SMV-PV [5], SMV [6]). Importantly, unlike our model, these earlier studies have been limited to detecting the presence or absence of tumor-vessel contact without quantifying the extent of vascular involvement. However, simply detecting tumor-vessel contact is insufficient for determining PDAC resectability, which limits the clinical relevance of these methods. Additionally, previous studies have only included patients with (borderline) resectable PDAC, whereas especially locally advanced PDAC is most challenging in daily clinical practice. Furthermore, these previous studies have often restricted the location of the tumors included in their analysis (*e.g.*, excluding tumors in the tail [4], including only tumors in the head [6], or head and uncinate process [5]). Our study considered PDAC tumors regardless of location, resectability, and preoperative systemic therapy. The size of our training set (613 CTs) is significantly larger than the data sets used in the previous four studies, which included 107 CTs [7], 194 CTs [4], 181 CTs [6], and 146 CTs [5]. Another key difference between this and previous studies is the reliance of these previous methods on manual segmentations to extract radiomic features. Besides manual segmentations being labor-intensive and prone to inter-observer variability, radiomic features are also more difficult to interpret than degree quantification, especially since degree quantification is part of the currently established radiological workflow and the primary determinant for tumor resectability. Quantification of degrees of vascular involvement is easier to interpret than radiomic features, especially given that any method trained on radiomic features for determining PDAC resectability is tied to the specific resection guidelines used for the training cases and becomes invalid if these guidelines change. In this case, the entire model would need to be retrained to adapt to the new guidelines, including the reevaluation of all training cases. In contrast, our model can easily be adapted to fit any resection guideline by simply changing the rules during the second step of our model and does not require any reevaluation of training cases.

There are several limitations to consider when interpreting our research. First, the ground-truth labels provided by the radiologist are labeled in categories (*e.g.*, 0–90°), which can complicate comparison with the precise calculations provided by our model. Second, radiologists will also consider additional anatomical factors, such as stenosis and occlusion of the vessel (part of the NCCN PDAC resectability guidelines [18]), which are currently not (yet) included in our model. Both stenosis and occlusion of a vessel should result in an automatic assessment of 360-degree involvement, even if the tumor-vessel contact appears to be less when analyzed geometrically using a CT scan. Third, we utilized resectability assessments from one of three radiologists as the ground truth, rather than a consensus among all three, which could introduce a level of subjectivity and potential bias in our findings. This approach may limit the generalizability and robustness of our results, as it does not account for inter-rater variability. Fourth, the method we used to quantify tumor-vessel contact relies on the vessel’s circumference rather than following a centerline approach, which can result in inaccurate results in cases of vascular deformation. Fifth, while our model performed well for arterial involvement, it performed worse for venous involvement. Upon further investigation, we discovered that the performance discrepancy was due to the quality of our AI segmentations. Specifically, our teacher segmentation model, which was only trained on CT-LAPs, demonstrated poorer performance on venous segmentations (SMV and PV) than arterial segmentations (CeTr, HA, SMA). In some cases, our teacher segmentation model failed to accurately identify the SMV, particularly in the tumor’s proximity, resulting in reduced accuracy for quantifying the tumor-vessel relationship. The main strength of our study is that the assessment of vascular involvement and tumor resectability is automatic and explainable, as it follows the existing radiological workflow, which can facilitate the integration of the model into routine clinical practice. Future research should incorporate CT-PVPs in their segmentation models to facilitate more accurate venous segmentations and, subsequently, improved assessment of vein-tumor interactions. Additionally, incorporating more cases of locally advanced PDAC during the training of the teacher segmentation model could also improve SMV segmentations, given the critical role of these veins in locally advanced PDAC and their tendency to be poorly visible, particularly in the arterial phase due to decreased blood flow. Furthermore, future work should incorporate stenosis and occlusion detection as additional factors for evaluating vascular involvement and adopt a more complex centerline approach for the geometric analysis of tumor-vessel contact. In addition, developing pathology-validated ground truth labels for vascular involvement, particularly in distinguishing fibrosis from tumor tissue, would be valuable for future research. Similarly, it is paramount for clinical implementation to evaluate the model against multiple raters, as this would provide a more rigorous and comprehensive measure of the system’s accuracy and reliability. Finally, a prospective study is needed to validate the performance of our model.

In conclusion, we developed a fully automatic AI-based model for segmenting PDAC tumors and surrounding vasculature, quantifying vascular involvement, and classifying resectability. Our results suggest that our model may allow automatic quantification of vascular involvement and classification of resectability for PDAC.

### Supplementary Information

**Additional file 1: Table S1.** Dutch Pancreatic Cancer Group criteria for PDAC resectability  [16]. **Table S2.** Distribution of vascular involvement per vessel in the test set comprising 60 PDAC patients.

## Data Availability

The documented code for the current study is available in the Vessel-Involvement-Quantifier repository (https://github.com/PHAIR-Consortium/Vessel-Involvement-Quantifier). Raw CT data and segmentation masks used and/or analyzed during the current study are available from the corresponding author upon reasonable request.

## Abbreviations

AI: Artificial intelligence
CeTr: Celiac trunk
CT: Computed tomography
CT-LAP: Late arterial phase CT
CT-PVP: Portal-venous phase CT
DPCG: Dutch Pancreatic Cancer Group
HA: Hepatic artery
LAPC: Locally advanced PDAC
PDAC: Pancreatic ductal adenocarcinoma
PV: Portal vein
SMA: Superior mesenteric artery
SMV: Superior mesenteric vein
