# Supplementary material for: Artificial intelligence for assessment of vascular involvement and tumor resectability on CT in patients with pancreatic cancer
Source: Eur Radiol Exp. 2024 Feb 12;8:18. doi: 10.1186/s41747-023-00419-9 (PMC10859357; doi:10.1186/s41747-023-00419-9)
Supplement: Supplementary file 1 — Additional file 1: Table S1. Dutch Pancreatic Cancer Group criteria for PDAC resectability [16]. Table S2. Distribution of vascular involvement per vessel in the test set comprising 60 PDAC patients. [file 41747_2023_419_MOESM1_ESM.docx]

**Artificial intelligence for assessment of vascular involvement and tumor resectability on CT in patients with pancreatic cancer**

**ELECTRONIC SUPPLEMENTARY MATERIAL**

**Segmentation Process in 3D Slicer**

The segmentation process in 3D Slicer commenced with the loading of the DICOM files, where the most suitable phase, preferably late arterial, was chosen. Subsequently, window settings were adjusted to CT abdomen parameters. New segments were then created in the Segment Editor tab and populated using the paintbrush function. Corrections were made as needed using the eraser function. To expedite and improve accuracy in segmenting structures like the kidneys, editable intensity ranges were set based on appropriate Hounsfield Unit (HU) values. Upon completion of all segmentations, both the scan and the segmentation data were exported in the NIfTI (.nii.gz) file format.

**Table S1:** Dutch Pancreatic Cancer Group criteria for PDAC resectability.[16]

| **Category** | **Celiac trunk** | **Hepatic artery** | **Superior mesenteric artery** | **Superior mesenteric vein** | **Portal vein** |
| --- | --- | --- | --- | --- | --- |
| Resectable | 0° | 0° | 0° | 0 – 90 ° | 0 – 90° |
| Borderline resectable | 0 – 90 ° | 0 – 90 ° | 0 – 90 ° | 0 – 270 ° | 0 – 270 ° |
| Locally advanced | 90 – 360 ° | 90 – 360 ° | 90 – 360 ° | 270 – 360 ° | 270 – 360 ° |

**Table S2:** Distribution of vascular involvement per vessel in the test set comprising 60 PDAC patients.

| **Degrees of involvement** | **Celiac trunk** | **Hepatic artery** | **Superior mesenteric artery** | **Superior mesenteric vein** | **Portal vein** | |
| --- | --- | --- | --- | --- | --- | --- |
| 0 – 90 | 55 | 49 | 48 | 31 | 42 | |
| 90 – 180 | 0 | 2 | 4 | 18 | 12 | |
| 180 – 270 | 1 | 2 | 2 | 4 | 2 | |
| 270 – 360 | 4 | 7 | 6 | 7 | 4 | |
|  | | | | | |  |
